# Tie2 Signaling Enhances Mast Cell Progenitor Adhesion to Vascular Cell Adhesion Molecule-1 (VCAM-1) through α4β1 Integrin

**DOI:** 10.1371/journal.pone.0144436

**Published:** 2015-12-11

**Authors:** Kazumasa Kanemaru, Emiko Noguchi, Takahiro Tokunaga, Kei Nagai, Takashi Hiroyama, Yukio Nakamura, Satoko Tahara-Hanaoka, Akira Shibuya

**Affiliations:** 1 Department of Immunology, Faculty of Medicine, University of Tsukuba, Tsukuba, Ibaraki, Japan; 2 Department of Medical Genetics, Faculty of Medicine, University of Tsukuba, Tsukuba, Ibaraki, Japan; 3 Life Science Center of Tsukuba Advanced Research Alliance (TARA), University of Tsukuba, Tsukuba, Ibaraki, Japan; 4 Graduate School of Comprehensive Human Sciences, University of Tsukuba, Tsukuba, Ibaraki, Japan; 5 Core Research for Evolutional Science and Technology (AMED-CREST), Tokyo, Japan; 6 Cell Engineering Division, RIKEN BioResource Center, Kounodai, Tsukuba, Ibaraki, Japan; 7 Department of Otorhinolaryngology Head and Neck Surgery, University of Fukui, Fukui, Japan; Institut Albert Bonniot-INSERMU823, FRANCE

## Abstract

Mast cell (MC) activation contributes considerably to immune responses, such as host protection and allergy. Cell surface immunoreceptors expressed on MCs play an important role in MC activation. Although various immunoreceptors on MCs have been identified, the regulatory mechanism of MC activation is not fully understood. To understand the regulatory mechanisms of MC activation, we used gene expression analyses of human and mouse MCs to identify a novel immunoreceptor expressed on MCs. We found that *Tek*, which encodes Tie2, was preferentially expressed in the MCs of both humans and mice. However, Tie2 was not detected on the cell surface of the mouse MCs of the peritoneal cavity, ear skin, or colon lamina propria. In contrast, it was expressed on mouse bone marrow–derived MCs and bone marrow MC progenitors (BM-MCps). Stimulation of Tie2 by its ligand angiopoietin-1 induced tyrosine phosphorylation of Tie2 in MEDMC-BRC6, a mouse embryonic stem cell-derived mast cell line, and enhanced MEDMC-BRC6 and mouse BM-MCp adhesion to vascular cell adhesion molecule-1 (VCAM-1) through α4β1 integrin. These results suggest that Tie2 signaling induces α4β1 integrin activation on BM-MCps for adhesion to VCAM-1.

## Introduction

Mast cells (MCs) are bone marrow (BM)–derived mononuclear cells, found in various tissues, such as the skin and mucosae, that function as sentinel cells in response to pathogens or other signs of infection [1]. Conversely, MCs are also associated with pathological conditions such as allergy through their production of proteases, vasodilating substances, cytokines, and lipid mediators [2].

Numerous studies have reported that the number of MCs increases at the inflammatory sites of allergic diseases in humans and allergic disease models in mice [3–5], probably as a result of the recruitment of MC progenitors (MCp) to those sites [6–9]. Like other leukocytes, MCp recruitment to peripheral tissues is regulated by integrins expressed on MCps [6,10]. To migrate across vascular endothelial cells (ECs) into peripheral tissues, MCps require the α4β1 and α4β7 integrins on their surface to bind to their ligands, mucosal addressin cell adhesion molecule-1 (MAdCAM-1) and vascular cell adhesion molecule-1 (VCAM-1), on the vascular ECs [7,11–13]. Especially in inflamed tissues, such as lung and skin, MCp binding to VCAM-1 but not to MAdCAM-1 is essential for MCp transmigration [7,12]. However, the role of chemokine receptors on MCps remains obscure [14,15], and the mechanism of integrin activation on MCps is not fully understood.

After recruitment to the inflammatory site, several cell surface receptors promote MC activation to enhance immune responses [16]. The high-affinity Fc receptor for IgE (FcεRI) has a critical role in MC activation [17,18]. In addition, other receptors on MCs, such as Toll-like receptors, cytokine receptors, complement receptors, and purinergic receptors recognize the signs of inflammation and transduce activating signals in MCs [16,19]. To regulate MC activation, the MCs also express inhibitory receptors on their cell surface [20–24]. However, the regulatory mechanisms of MC activation remain incompletely understood.

Furthermore, even though various receptors that are involved in MCp recruitment and MC activation have been identified on the cell surface of MCps and MCs, there are still no effective strategies to regulate these events through modulation of the receptor functions for the treatment of allergic diseases. For these reasons, we sought to identify a novel signal-transducing receptor on MCps or MCs that regulates MCp or MC activation.

## Methods

### Human samples

Peripheral blood mononuclear cells were isolated from the blood of healthy volunteers. Written informed consents were obtained from the volunteers. This study was approved by the ethical review boards of the University of Tsukuba.

### Mice

C57BL/6 mice were purchased from Clea Japan (Tokyo, Japan). All mice used were 8–12-week-old females or males. All animal experiments in this study were carried out humanely after receiving approval from the Animal Ethics Committee of the Laboratory Animal Resource Center, University of Tsukuba, and in accordance with Fundamental Guideline for Proper Conduct of Animal Experiment and Related Activities in Academic Research Institutions under the Jurisdiction of the Ministry of Education, Culture, Sports, Science and Technology.

### Cells

Human peripheral blood–derived cultured MCs (PB-MCs) were generated, as described [22]. Human T cells (CD3^+^), B cells (CD19^+^), and monocytes (CD14^+^) were isolated from human peripheral blood mononuclear cells by using a MACS cell separation system (Miltenyi Biotec, Bergisch Gladbach, Germany).

Mouse BM–derived cultured MCs (BMMCs) were generated, as described [25].

Mouse ear skin cells were isolated as previously described [26] with a minor modification. In brief, ear tissue was minced, resuspended in RPMI 1640 medium containing 10% FBS and 400 U/mL collagenase type 2 (Worthington Biochemical Corporation, Lakewood, NJ), incubated at 37°C for 60 min in an orbital shaker, and passed through nylon wool mesh. Cells were then analyzed by flow cytometry.

Mouse colon lamina propria cells were isolated, as previously described [27] with a minor modification. In brief, colon tissues were opened longitudinally, minced into 5 to 10 mm pieces, and washed extensively with cold PBS. Mucosal pieces were incubated twice with 5 mM EDTA (Sigma-Aldrich, St. Louis, MO) in Hanks’ balanced salt solution (HBSS) (Sigma-Aldrich) for 30 min at 37°C, washed with cold PBS and then incubated for 50 min in HBSS containing 400 U/ml collagenase type 2 and 0.1 mg/mL DNase I (Worthington Biochemical Corporation). Large debris was removed from the cell suspension by passage through nylon wool mesh. Cells were isolated by using Percoll density gradient centrifugation (GE Healthcare Biosciences, Little Chalfont, U.K.), and analyzed by flow cytometry.

The mouse embryonic stem cell–derived mast cell line MEDMC-BRC6 was generated at RIKEN BioResource Center (Tsukuba, Japan) [28].

### RNA sequencing (RNA-seq)

Total RNA of human PB-MCs, T cells, B cells, and monocytes were extracted by using an RNeasy Mini kit (Qiagen, Hilden, Germany), and a Ribo-Zero rRNA Removal Kit (Illumina, San Diego, CA) was used to remove ribosomal RNA. RNA-seq was performed according to the protocol described in the SOLiD Total RNA-Seq Kit (Life Technologies, Carlsbad, CA). The library was subjected to emulsion PCR (SOLiD™ EZ Bead™ Emulsifier kit, Life Technologies) to generate clonal DNA fragments on beads, followed by bead enrichment (SOLiD™ EZ Bead™ Enrichment kit, Life Technologies). Enriched template beads were sequenced on a SOLiD 5500xl sequencer as single-end, 75-bp reads (Life Technologies). The SOLiD 5500xl output reads were aligned against the human genome reference sequence (hg19) by using LifeScope version 2.5.1 (Life Technologies) to generate BAM files, and subsequent data analysis was performed in Avadis NGS (Strand Scientific Intelligence Inc., San Francisco, CA). The RNA-seq dataset generated in this study was deposited in NCBI's Gene Expression Omnibus under the accession number GSE71247.

### Gene data analyses

To select genes encoding immunoglobulin (Ig)-like receptors or C-type lectin/C-type lectin-like (CLECT) receptors, and immunoreceptors containing signaling motif sequences or catalytic domains in their intracellular portion, we analyzed the predicted amino acid sequences of genes expressed in human PB-MCs by using the NCBI conserved domain database [29] and in-house Perl scripts. The amino acid sequences of signaling motifs are shown in S1 Table. To analyze gene expression in mouse MCs, we used the microarray data (GSE10246). Gene expression values in MCs were defined as the maximum value of the MC samples in GSE10246. The sample names in GSE10246 are shown in S2 Table.

To analyze the extent of specific gene expression in human MCs, data from our RNA-seq analysis were used. The extent of specific gene expression was calculated as the average of each fold change (FC) in gene expression between the MCs and the other cell types (T cells, B cells, and monocytes).

To analyze the extent of specific gene expression in mouse MCs, data from GSE10246 were used. Gene expression for each cell type was defined as the maximum value of the samples of each cell type (S2 Table). The extent of specific gene expression in mouse MCs was calculated in the same manner as for the human data.

### Antibodies

Phycoerythrin (PE)/cyanine (Cy) 7-conjugated anti-human c-Kit (104D2) monoclonal antibody (mAb), allophycocyanin (APC)/Cy7-conjugated anti-mouse CD8a (53–6.7), CD11b (M1/70), and B220 (RA3-6B2) mAbs, APC/H7-conjugated anti-mouse CD4 (GK1.5) mAb, BD Horizon V450-conjugated anti-mouse Gr-1 (RB6-8C5) mAb, Pacific blue-conjugated anti-mouse CD3e (500A2) mAb, biotin-conjugated anti-mouse CD4 (GK1.5), CD8a (53–6.7), CD11b (M1/70), B220 (RA3-6B2), and TCRβ (H57-597) mAbs, unconjugated anti-β1 integrin (Ha2/5 and 9EG7) mAbs, and fluorescein-conjugated streptavidin were purchased from BD Biosciences (San Jose, CA). Fluorescein isothiocyanate (FITC)-conjugated anti-mouse CD27 (LG.3A10) and Sca-1 (D7) mAbs, PE-conjugated anti-human Tie2 (33.1) and anti-mouse β7 integrin (FIB27) mAbs, PE/Cy7-conjugated anti-mouse c-Kit (2B8) mAb, APC-conjugated anti-mouse ST2 (D1H9) mAb, PerCP/Cy5.5-conjugated anti-mouse β7 integrin (FIB27) mAb, biotin-conjugated anti-mouse TER-119 (TER-119) and Tie2 (TEK4) mAbs, and unconjugated anti-β7 integrin (FIB27) mAb were purchased from BioLegend (San Diego, CA). FITC-conjugated anti-mouse FcεRIα (MAR-1) mAb, and biotin-conjugated anti-mouse FcεRIα (MAR-1) mAb were purchased from eBioscience (San Diego, CA). Biotin-conjugated anti-mouse Gr-1 (RB6-8C5) mAb was purchased from Beckman Coulter (Pasadena, CA). Horseradish peroxidase (HRP)–conjugated anti-phosphotyrosine (pTyr) Ab (4G10 platinum) was purchased from Merck Millipore (Darmstadt, Germany). Unconjugated anti-Flag (M2) mAb and anti-Flag polyclonal Ab were purchased from Sigma-Aldrich. HRP-conjugated anti rabbit IgG Ab was purchased from GE Healthcare Biosciences.

### Flow cytometry

Flow cytometric analyses and cell sorting were performed by using FACS LSRFortessa and FACS Aria flow cytometers (BD Biosciences), respectively. FlowJo software (Tree Star, Ashland, OR) was used for data analyses. Dead cells were stained and excluded by using Propidium iodide solution (P4864, Sigma-Aldrich) or Zombie Violet Fixable Viability Kit (423114, BioLegend).

### Isolation of mouse BM-MCp

Mouse BM-MCps were isolated, as previously described [30]. For analyses of Tie2 expression on BM-MCps, BM cells were stained with anti-lineage mAb cocktail (including Pacific blue-conjugated anti-CD3 mAb, BD Horizon V450-conjugated anti-Gr-1 mAb, and APC/Cy7-conjugated anti-CD4, CD8, CD11b, and B220 mAbs), PE/Cy7-conjugated anti-c-Kit mAb, FITC-conjugated anti-FcεRIα, CD27, and Sca-1 mAbs, PerCP/Cy5.5-conjugated anti-β7 integrin mAb, and APC-conjugated anti-ST2 mAb. Tie2 expression on BM-MCps was analyzed by flow cytometry (FACS LSRFortessa flow cytometer, BD Biosciences) with biotin-conjugated anti-Tie2 mAb, followed by PE-conjugated streptavidin.

For BM-MCp isolation, BM cells were stained with biotin-conjugated mAbs specific for lineage markers: TCRβ, CD4, CD8, B220, CD11b, Gr-1, and TER-119. Cells were then incubated with Streptavidin-Particle Plus-DM (BD biosciences). Lineage positive cells were removed by BD IMagnet (BD biosciences). The remaining cells were stained with FITC-conjugated anti-Sca-1 and CD27 mAbs, PE-conjugated anti-β7 integrin mAb, PE/Cy7-conjugated anti-c-Kit mAb, APC-conjugated anti-ST2 mAb, and biotin-conjugated mAbs (anti-TCRβ, CD4, CD8, B220, CD11b, Gr-1, and TER-119) specific to lineage markers and biotin-conjugated anti-FcεRIα mAb, followed by APC/Cy7-conjugated streptavidin. BM-MCps (lineage^−^ c-Kit^+^ FcεRIα^−^ Sca-1^−^ CD27^−^ β7 integrin^+^ ST2^+^) were sorted by flow cytomery (FACS Aria flow cytometer, BD Biosciences).

### Complementary DNA synthesis and real-time (RT)-PCR

Total RNA was extracted with Isogen reagent (Nippon Gene, Tokyo, Japan), and cDNA was synthesized by using a High Capacity RNA-to-cDNA Kit (Applied Biosystems, Carlsbad, CA). *Tek* expression was measured with quantitative RT-PCR, performed with SYBR Green master mix (Applied Biosystems) and the specific primers. The *Gapdh* expression level was used as an internal control to normalize data. Primer sequences of the target genes are: *Tek*, forward, 5’-GTGAAGGTCGAGTTCGAGGA-3’, reverse, 5’-CCCTGTCCACGGTCATAGTT-3’; *Gapdh*, forward, 5’-TGGTGAAGGTCGGTGTGAAC-3’, reverse, 5’-ATGAAGGGGTCGTTGATGGC-3’.

### Establishment of transfectants

MEDMC-BRC6 transfectants stably expressing wild-type (WT) Tie2 or mutant Tie2 lacking the cytoplasmic portion (ΔCyt) and tagged with a Flag at the N-terminus were established as previously described [25], by transfection with WT *Tek* cDNA and mutated *Tek* cDNA encoding the extracellular and transmembrane portions subcloned into the pMXs retroviral vector [31].

### Biochemical analysis

To analyze the tyrosine phosphorylation of Tie2, MEDMC-BRC6 transfectants were stimulated with recombinant human angiopoietin-1 (Ang1) (923-AN; R&D Systems, Minneapolis, MN) (250 ng/mL) for 3 to 10 min at 37°C, lysed with 1% NP-40 lysis buffer, and immunoprecipitated with an anti-Flag M2 mAb (F3165; Sigma-Aldrich). Immunoprecipitates were resolved by SDS–PAGE, transferred onto polyvinylidene difluoride membranes by electroblotting, immunoblotted with HRP-conjugated anti-pTyr Ab (4G10 and PY20; Merck Millipore) and an anti-Flag polyclonal Ab, followed by an HRP-conjugated anti-rabbit IgG Ab. Proteins were detected by enhanced chemiluminescence (Thermo Fisher Scientific, Waltham, MA).

### Adhesion assay

MEDMC-BRC6 transfectants (3 × 10^4^ per well), mouse BM-MCps (5 × 10^3^ to 1 × 10^4^ per well), or mouse BMMCs (3 × 10^4^ per well) were incubated in the presence or absence of recombinant human Ang1 (923-AN; R&D Systems) (250 ng/mL) with or without a neutralizing anti-β1 integrin mAb (Ha2/5) (20 μg/mL), a neutralizing anti-β7 integrin mAb (FIB27) (20 μg/mL), or a control Ab (hamster IgM, rat IgG2a) (20 μg/mL) for 30 min to 1 h. Cells were then cultured for 1 h in flat-bottomed 96-well plates that were precoated with a human IgG1 Ab (AG502; Merck Millipore) or mouse VCAM-1-Fc (643-VM; R&D Systems) (3 μg/mL) for 16 h and blocked for 1 h with PBS containing 2% BSA. After removal of the non-adherent cells by gentle washing with PBS, the number of adherent cells in 20 mm^2^ per well was counted under a BZ-X710 All-in One Fluorescence Microscope (Keyence, Osaka, Japan).

### Statistical analysis

Statistical analyses were performed by using the two-tailed Student’s t-test (GraphPad Prism 5, GraphPad Software, La Jolla, CA) for quantitative RT-PCR assay or the ANOVA test with the post-hoc Tukey-Kramer test (GraphPad Prism 5, GraphPad Software) for adhesion assays.

## Results

### Identification of *Tek* expression in MCs

**Fig 1:**
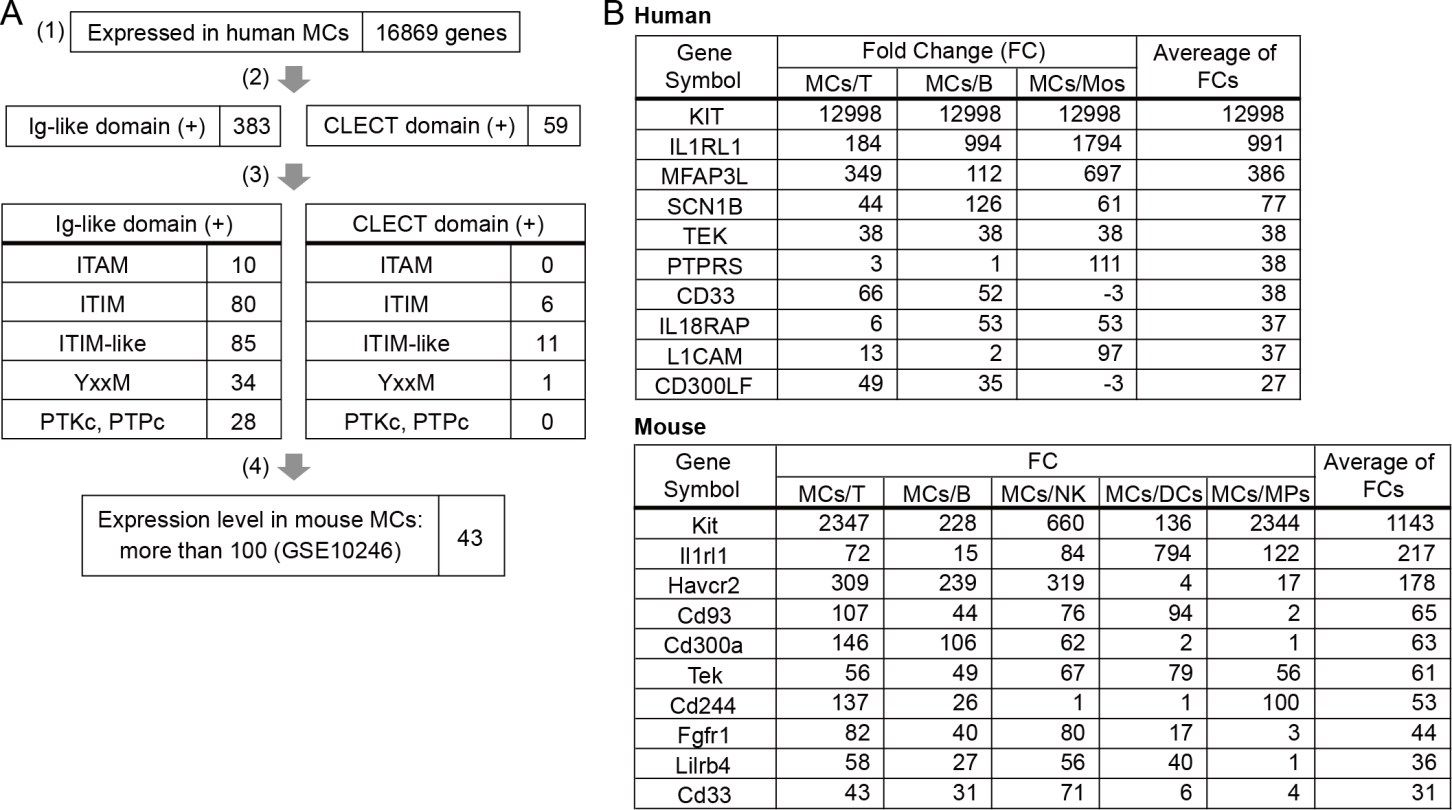
*Tek* expression in MCs. (A) Candidate genes were selected on the basis of human MC gene expression data obtained from RNA-seq analysis (1), the NCBI conserved domain database (2)(3), in-house Perl scripts (3), and mouse MC gene expression data obtained from the microarray data (GSE10246) (4). (B) The expression of selected genes in MCs were compared with that in other cell types by using human (GSE71247) and mouse (GSE10246) gene expression data. MCs, mast cells; T, T cells; B, B cells; Mos, monocytes; NK, natural killer cells; DCs, dendritic cells; MPs, macrophages.

To identify a novel receptor that regulates MC activation, we performed RNA-seq analysis of human MCs, which were induced by culture of CD34^+^ hematopoietic stem cells (HSC) in peripheral blood [22,32]. Human peripheral blood-derived MCs (PB-MCs) were found to express 16,869 genes. By using the NCBI conserved domain database [29] to analyze the predicted amino acid sequences, we selected 383 and 59 genes encoding proteins that belong to the Ig-like receptor superfamily and the CLECT receptor family, respectively (Fig 1A). We then used in-house Perl scripts and the NCBI conserved domain database to select genes encoding receptors that potentially mediated activating or inhibitory signals through the amino acid sequences (S1 Table) of following signaling motifs or catalytic domains in their intracellular regions: immunoreceptor tyrosine-based activation motif (ITAM), immunoreceptor tyrosine-based inhibitory motif (ITIM) or ITIM-like amino acid sequences, PI3K binding motif, or conserved catalytic domains of protein tyrosine kinases (PTKc) and protein tyrosine phosphatase (PTPc) (Fig 1A, S3 Table). Next, we examined the gene expression levels of the candidates in mouse MCs by using the published microarray data (GSE10246) based on BMMC analysis, and selected genes with a normalized expression level of more than 100 (Fig 1A, S3 Table). Finally, to select genes preferentially expressed in MCs compared with other cell types, we analyzed the extent of specific expression in MCs by using the RNA-seq data from human cells and the data from GSE10246 (Fig 1B). On the basis of our results, we focused on the Tie2-encoding gene *Tek*, which was expressed at higher levels in human and mouse MCs compared with other cell types.

### Tie2 is expressed on BMMCs and BM-MCps in mice and PB-MCs in humans

**Fig 2:**
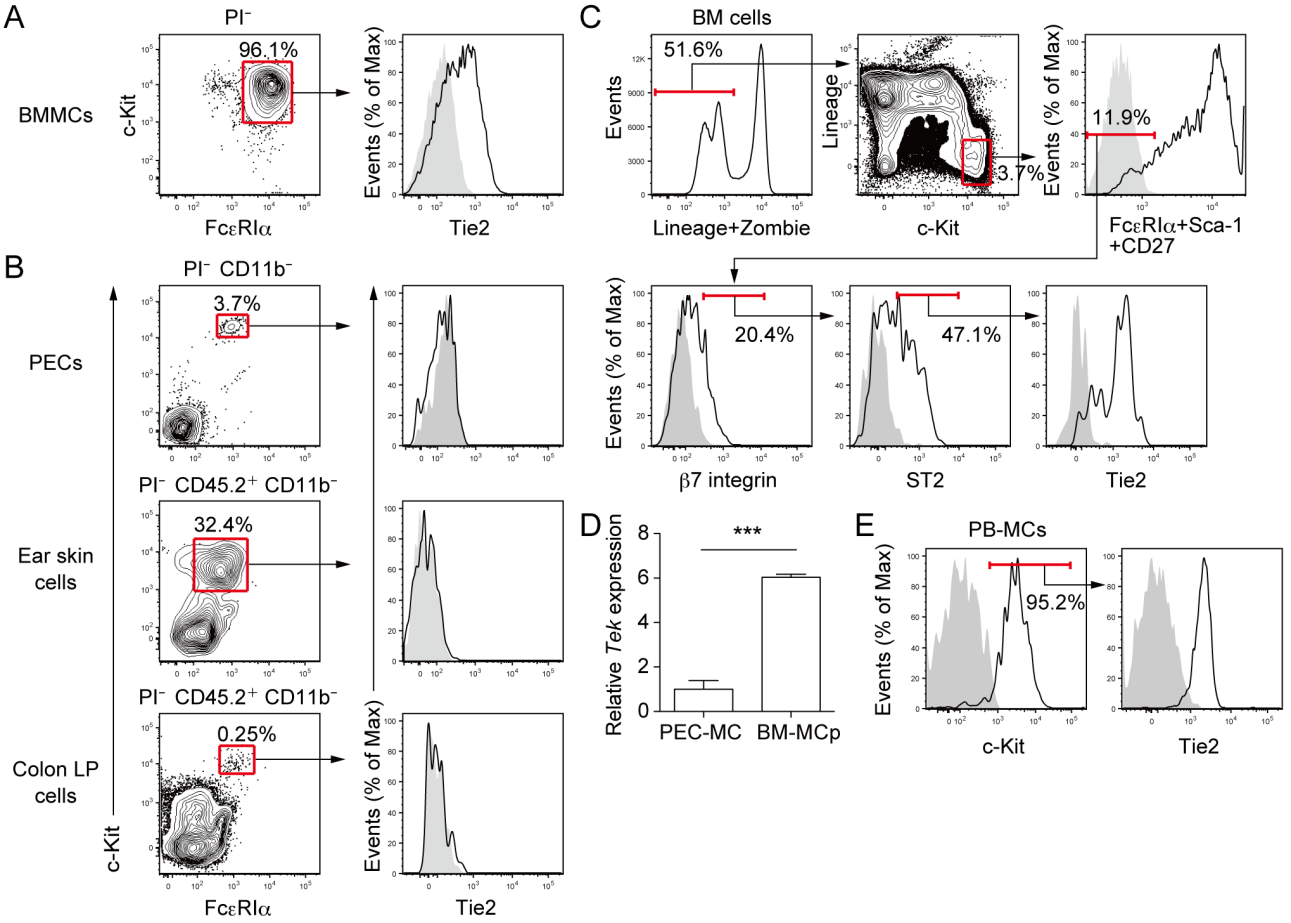
Tie2 is expressed on BMMCs and BM-MCps in mice and PB-MCs in humans. (A) Mouse BMMCs were generated as described in the Methods. Tie2 expression on BMMCs was analyzed by staining with an isotype control Ab and an anti-mouse Tie2-specific mAb. Stained cells were analyzed by using flow cytometry. (B) MCs in mouse peritoneal exudate cells (PECs), ear skin cells, and colon lamina propria (LP) cells were detected by using the Abs described in the Methods. Tie2 expression on each cell was analyzed as described in A. (C) Mouse BM-MCps were detected as described in the Methods. Tie2 expression on BM-MCps was analyzed as described in A. Lineage markers: CD3, CD4, CD8, CD11b, B220, and Gr-1. (D) Mouse BM-MCps were sorted as described in the Methods. Mouse PEC-MCs were sorted by using the Abs described in the Methods and flow cytometry. *Tek* expression was measured by using real-time reverse transcription-PCR. (E) Human PB-MCs were generated as described in the Methods, and Tie2 expression was analyzed by flow cytometry. Shaded histograms show staining of isotype control Ab. Data show mean values ± SEM (n = 5). ***p < 0.001.

Next, we analyzed Tie2 expression on the cell surface of mouse MCs. Although BMMCs expressed Tie2 on the cell surface (Fig 2A), Tie2 was not expressed on MCs in the peritoneal cavity, ear skin, or colon lamina propria (Fig 2B). Since BMMCs are considered immature compared with tissue-resident MCs, on the basis of their granule contents [33,34], we hypothesized that MCps in the bone marrow (BM-MCps) might also express Tie2. As predicted, Tie2 was expressed on BM lineage^−^ c-Kit^+^ FcεRIα^−^ Sca-1^−^ CD27^−^ β7 integrin^+^ ST2^+^ cells (Fig 2C) that has been defined as BM-MCp population [30]. In addition, *Tek* expression was detected in sorted BM-MCps, and it was significantly higher than that in sorted MCs of peritoneal cavity (Fig 2D). We found that Tie2 was also expressed on human PB-MCs, which were characterized by c-Kit^+^ cells after the culture of CD34^+^ HSCs in the presence of SCF, IL-6, and IL-3 [22,32] (Fig 2E).

### Tie2 signaling enhances MEDMC-BRC6 cell adhesion to VCAM-1 through α4β1 integrin

**Fig 3:**
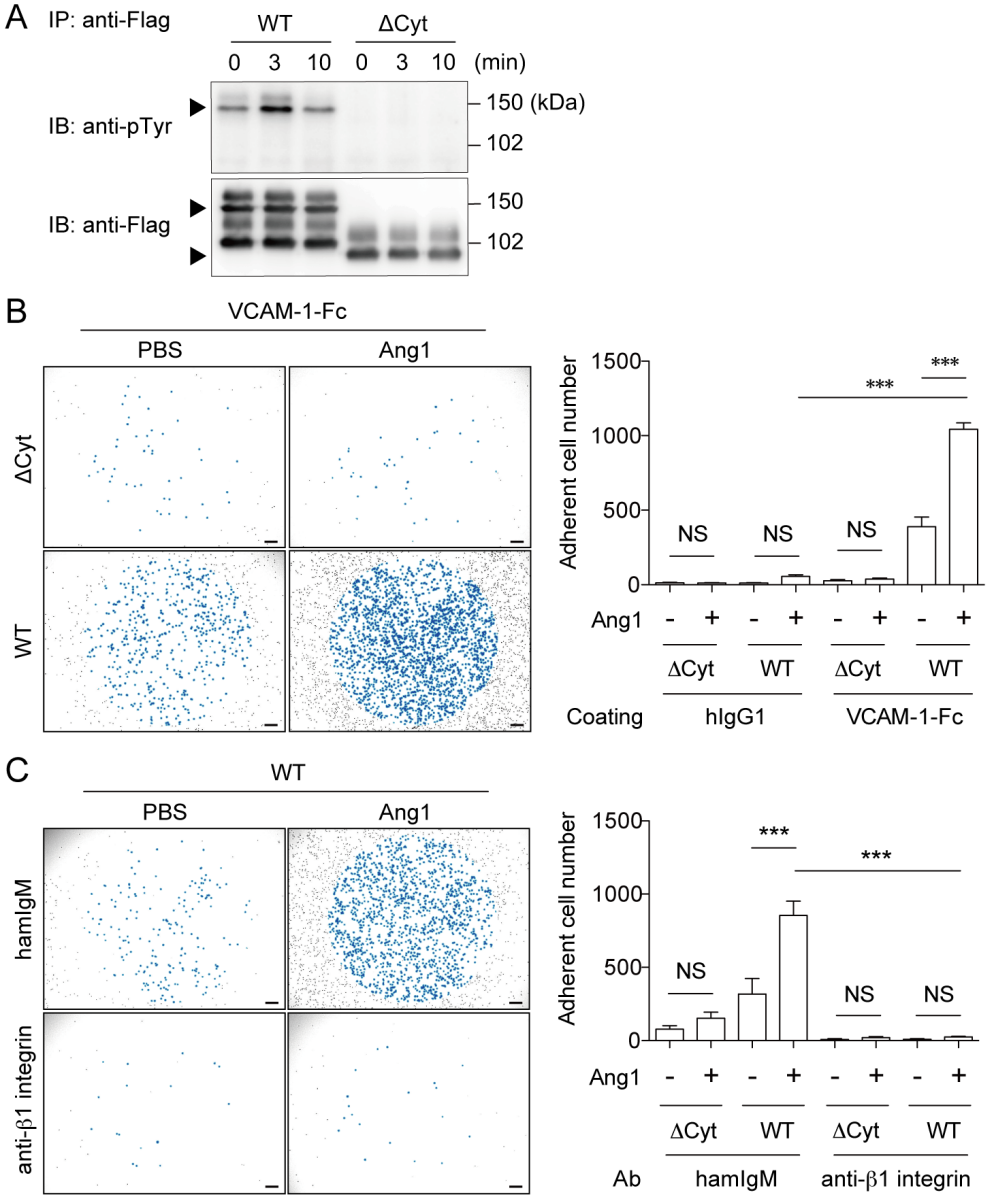
Tie2 signaling enhances MEDMC-BRC6 adhesion to VCAM-1 through α4β1 integrin. (A) WT/MEDMC-BRC6 and ΔCyt/MEDMC-BRC6 transfectants were established as described in the Methods. Transfectants were stimulated with Ang1 (250 ng/mL), and immunoprecipitated (IP) with an anti-Flag mAb. Tyrosine phosphorylation of Tie2 was analyzed by blotting with anti-pTyr Ab. (B) WT/MEDMC-BRC6 and ΔCyt/MEDMC-BRC6 were treated with Ang1 (250 ng/mL) or not and incubated on hIgG1- or VCAM-1-Fc-coated wells. Adherent cells, as observed blue-colored, were counted by using a microscope (20 mm^2^ per well). (C) Neutralizing anti-β1 integrin Ab (20 μg/mL) or control Ab (20 μg/mL) was added under the conditions described in B. Cells were incubated on VCAM-1-Fc-coated wells, and adherent cells were similarly counted. WT, WT/MEDMC-BRC6; ΔCyt, ΔCyt/MEDMC-BRC6. Scale bars, 200 μm. Data show mean values ± SEM (n = 3 or 5). ***p < 0.001.

To study the function of Tie2 on MCps, we established MEDMC-BRC6 transfectants, which express either WT Tie2 (WT/MEDMC-BRC6) or Tie2 lacking the cytoplasmic portion (ΔCyt/MEDMC-BRC6). Cell surface expression of Tie2 was comparable between WT/MEDMC-BRC6 and ΔCyt/MEDMC-BRC6 (S1 Fig). After treatment of the transfectants with angiopoietin-1 (Ang1), which is an agonistic ligand of Tie2 [35], tyrosine phosphorylation of Tie2 was upregulated in WT/MEDMC-BRC6, but not in ΔCyt/MEDMC-BRC6 (Fig 3A). These results indicate that Ang1 induces Tie2 signaling in MEDMC-BRC6.

In inflammatory conditions such as allergic diseases, MCps migrate across vascular ECs into the inflamed tissue via the interaction of α4β1 and α4β7 integrins on MCps with VCAM1 on ECs [7,11,12]. Yet, Ang1-Tie2 signaling enhances cell adhesion to extracellular matrix via β1 integrin [36–38]. In addition, since Ang1 is expressed by peri-endothelial mural cells [39], we hypothesized that Ang1-Tie2 signaling in MCps may participate in MCp migration across vascular ECs by regulating MCp adhesion to VCAM-1 via α4β1 integrin. To test this hypothesis, we treated WT/MEDMC-BRC6 and ΔCyt/MEDMC-BRC6 with Ang1, both of which expressed α4 integrin and β1 integrin (S1 Fig), and examined the adhesion of these cells to plate-coated VCAM-1. We found that treatment with Ang1 enhanced MEDMC-BRC6 adhesion to VCAM-1, which required the cytoplasmic portion of Tie2 (Fig 3B). We then assessed the involvement of α4β1 integrin in this enhancement of adhesion. The addition of a neutralizing Ab against β1 integrin into this assay completely abolished the effect of Ang1-Tie2 signaling on MEDMC-BRC6 adhesion to VCAM-1 (Fig 3C). These results indicate that Tie2 signaling is involved in α4β1 integrin-mediated adhesion of MEDMC-BRC6 to VCAM-1.

We used two clones of anti-β1 integrin mAb (clone Ha2/5 and 9EG7) to analyze the β1 integrin expression on the transfectants (S1 Fig). Clone Ha2/5 recognizes both active and inactive form of β1 integrin, and clone 9EG7 recognizes an activation-associated epitope of β1 integrin. The binding of clone 9EG7 was downregulated in ΔCyt/MEDMC-BRC6 compared with WT/ MEDMC-BRC6 (S1 Fig). In contrast, the downregulation of the binding of clone Ha2/5 in ΔCyt /MEDMC-BRC6 was markedly less, compared with that of clone 9EG7 (S1 Fig). These results indicate that the difference of clone 9EG7 binding between WT and ΔCyt/MEDMC-BRC6 was due to less active form of β1 integrin on ΔCyt/MEDMC-BRC6.

### Ang1 treatment enhances mouse BM-MCp adhesion to VCAM-1 through α4β1 integrin

**Fig 4:**
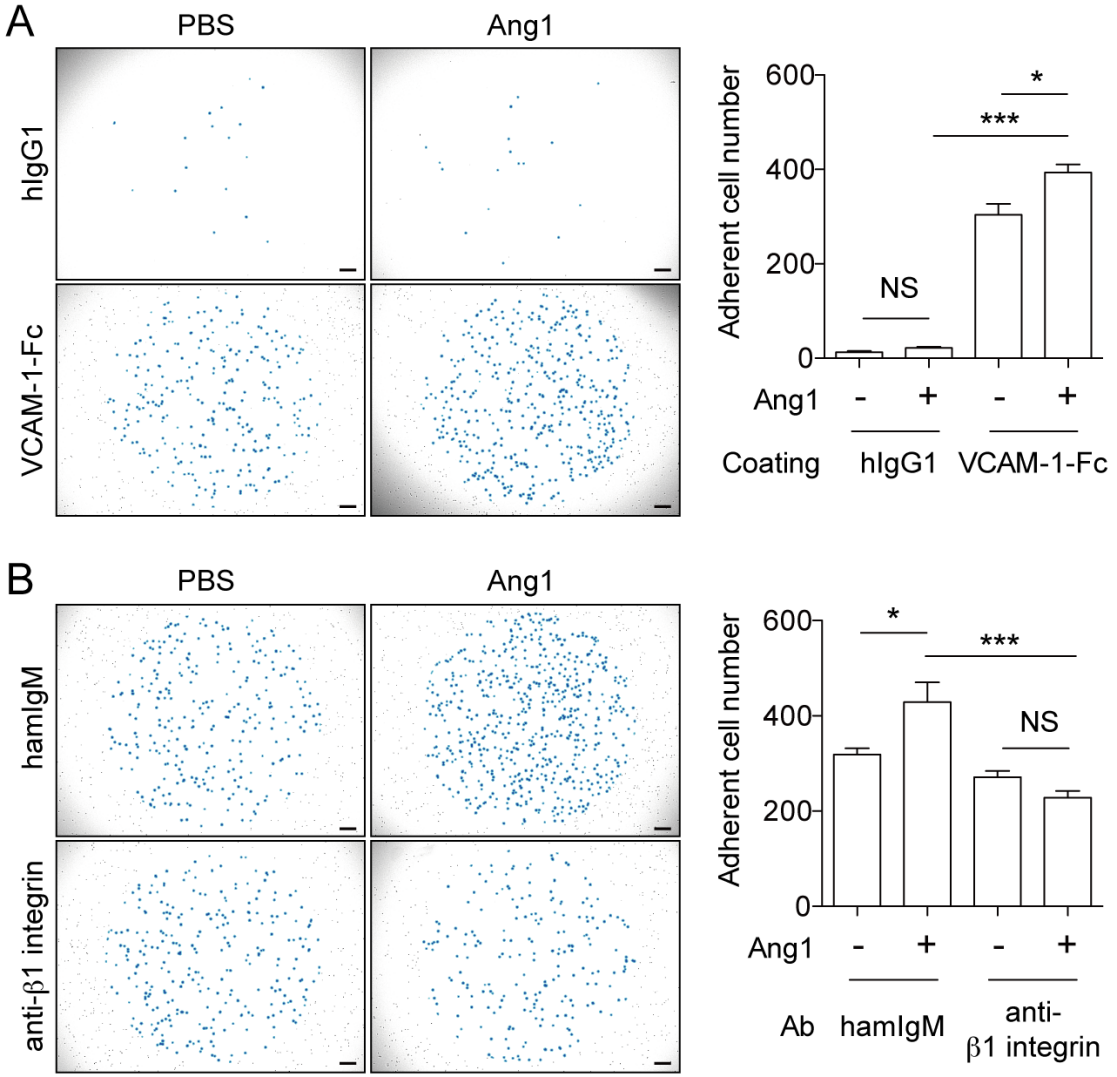
Ang1 treatment enhances mouse BM-MCp adhesion to VCAM-1 through α4β1 integrin (A) Mouse BM-MCps were stimulated with or without Ang1 (250 ng/mL) and cultured in wells that were precoated with human IgG1 Ab or mouse VCAM-1-Fc. Adherent cells, as observed blue-colored, were counted by using a microscope (20 mm^2^ per well). (B) Neutralizing anti-β1 integrin Ab (20 μg/mL) or control Ab (20 μg/mL) was added under the conditions described in A. BM-MCps were incubated on VCAM-1-Fc-coated wells, and adherent cells were similarly counted. Scale bars, 200 μm. Data show mean values ± SEM (n = 3 or 5). *p < 0.05, ***p < 0.001.

To examine whether Tie2 expressed on MCps regulates adhesion of MCps to VCAM-1, we sorted mouse BM-MCps from BM by means of flow cytometry. Similarly to MEDMC-BRC6 transfectant, while BM-MCps showed enhanced adhesion to plate-coated VCAM-1 after treatment with Ang1 (Fig 4A), the Ang1-induced enhancement of BM-MCp adhesion to VCAM-1 was completely abolished in the presence of a neutralizing Ab against β1 integrin (Fig 4B). These results indicate that Tie2 signaling is involved in the α4β1 integrin-mediated adhesion of MCps to VCAM-1.

It is reported that Ang1-Tie2 signaling enhances β1 integrin expression in HSCs and neoplastic glial cells [37,38]. To examine whether Ang1 treatment enhances β1 integrin expression or activation in MEDMC-BRC6 transfectants and BM-MCps, we used two clones of anti-β1 integrin mAbs (Ha2/5 and 9EG7) as described at the previous sub-section. As a result, binding of both mAbs against β1 integrin to MEDMC-BRC6 transfectants and BM-MCps were not enhanced after Ang1 treatment (S2 Fig). These results indicate that Ang1 treatment did not upregulate the expression of β1 integrin on the cell surface.

Unlike the adhesion of MEDMC-BRC6 transfectants, BM-MCp adhesion to VCAM-1 was not inhibited so extensively by adding a neutralizing Ab against β1 integrin. Since α4β7 integrin is also involved in VCAM-1 dependent MCp transmigration to peripheral tissue [7], we examined the involvement of β7 integrin in BM-MCp adhesion to VCAM-1 by adding a neutralizing anti-β7 integrin Ab into the adhesion assay. As a result, adding both anti-β1 integrin and anti-β7 integrin Abs inhibited BM-MCp adhesion to VCAM-1 greater than adding each of the Ab (S3 Fig). However, even in the condition that both neutralizing mAbs were added, BM-MCp adhesion to VCAM-1 was not inhibited completely. This result indicates that adhesion through integrins other than β1 and β7 integrin or other mechanisms are also involved in the BM-MCp adhesion to VCAM-1.

### Ang1 treatment enhances mouse BMMC adhesion to VCAM-1 through α4β1 and α4β7 integrin

To examine whether Ang1 treatment activate α4β7 integrin as well as α4β1 integrin for adhesion to VCAM1, we used mouse BMMCs, which, unlike MEDMC-BRC6 transfectants, expressed β7 integrin as well as β1 integrin (S1 and S4A Figs). Ang1 treatment enhanced BMMC adhesion to VCAM-1 (S4B Fig), and this effect was completely abolished by addition of a neutralizing Ab against β1 integrin (S4C Fig). However, addition of a neutralizing Ab against β7 integrin also significantly suppressed the effect of Ang1 treatment on the BMMC adhesion (S4D Fig). These results indicate that, although β1 integrin play a major role in BMMC adhesion to VCAM-1, β7 integrin is also involved in the adhesion induced by Ang1.

## Discussion

Tie2, a receptor-type tyrosine kinase, is expressed on vascular and lymphatic ECs, HSCs, and tumor-associated monocytes [39–41]. Ligands of Tie2 include Ang1 and angiopoietin-2 (Ang2) [39]. There is evidence that in both ECs and HSCs Ang1-Tie2 signaling promotes cell survival, migration, and cell adhesion to extracellular matrix such as fibronectin and collagen via integrin [36,39,42]. Although we found that *Tek* was preferentially expressed in MCs, it has remained unclear whether Tie2 is expressed on the cell surface of primary MCs and, if it is, what its functional role might be in MC activation.

We showed that although mouse BMMCs expressed Tie2, matured MCs in the mouse peripheral tissues did not (Fig 2A and 2B). The reason for this difference may be that the gene expression data from the human and mouse MCs used for the selection of Tie2 were obtained from *in vitro* differentiated MCs. In primary cells, we detected Tie2 expression on mouse BM-MCps in both protein and mRNA levels (Fig 3C and 3D). These results suggest that Tie2 expression on the cell surface is restricted to immature MCs and is suppressed after maturation. We found that Tie2 was also expressed on human PB-MCs (Fig 2E), which are immature MCs, consistent with the case of mouse immature MCs. In addition, lower expressions of *Tek* in mouse matured tissue MCs compared with the expression in hematopoietic progenitor cells can be also observed in the gene expression database (https://www.immgen.org).

We found that stimulation of Tie2 with Ang1 enhanced adhesion of MEDMC-BRC6 transfectants and mouse BM-MCps to VCAM-1 via α4β1 integrin (Figs 3 and 4). These results are consistent with previous reports that Ang1-Tie2 signaling enhances adhesion of HSCs and neoplastic glial cells to extracellular matrix via β1 integrin [36–38]. Moreover, there have been reports that the concentration of Ang1 is increased in the serum and lungs of patients with asthma [43,44]. These reports suggest that serum Ang1 may stimulate MCps to enhance their migration across vascular ECs into inflamed tissues. However, this concept is controversial, because another study reported a decrease, rather than an increase, in the serum titer of Ang1 in asthmatic patients [45]. The expression of Ang2, another ligand of Tie2, had also been reported to increase in asthmatic lung [43]. Ang2 is classically known to antagonize the Ang1-Tie2 interaction and to promote EC destabilization at the inflammatory site [46], whereas higher concentration of Ang2 have agonistic effects on Tie2 signaling and enhance EC survival [47,48]. Although further analyses are required, the role of the angiopoietin-Tie2 system in MCp transmigration is a promising target for the treatment of allergic diseases, by regulating the recruitment of MCps to inflamed tissues and preventing the increase in MC numbers at the site of inflammation.

The smaller effect of Ang1 treatment on BM-MCp adhesion to VCAM-1 may be due to a lower expression level of Tie-2 compared to WT/MEDMC-BRC6 transfectant. In addition, it may also be caused by the presence of Ang1 produced by stromal cells, including osteoblasts, and hematopoietic progenitor cells, in the BM [49,50], which had partially activated Tie2 on BM-MCps in the BM before isolation.

The expression of β1 integrin and the activation-associated epitope of β1 integrin that is recognized by clone 9EG7 were not enhanced by Ang1 treatment (S2 Fig). Since clone 9EG7 can recognize α4β1 integrin in the absence of the ligands [51,52], it may be possible that Ang1-Tie2 signaling enhances the transformation of α4β1 integrin from intermediate affinity form to high affinity form by promoting outside-in signaling after the interaction with VCAM-1.

Although Ang1 treatment of WT/MEDMC-BRC6 increased tyrosine phosphorylation of Tie2 and adhesion to VCAM-1, both tyrosine phosphorylation and adhesion were also detected even before the treatment at a lower level (Fig 3A and 3B). In addition, less active form of β1 integrin was expressed on ΔCyt/MEDMC-BRC6 than on WT/MEDMC-BRC6 (S1 Fig). These results may be caused by ligand-independent Tie2 dimerization in WT/MEDMC-BRC6, as reported in HEK293T transfectant expressing Tie2 showing tyrosine phosphorylation of Tie2 without any stimulation [53].

Ang-1 is also known to regulate HSC quiescence in BM hematopoietic niches [37]. In addition, hematopoietic stem and progenitor cells are retained in BM hematopoietic niches through the interaction between α4β1 integrin expressed on their surface and VCAM-1 present on cells that comprise the niches [54–56]. Thus, our data suggest that Ang-1 may also regulate BM-MCp retention in BM hematopoietic niches via Tie2 signaling.

To analyze Tie2 function more definitively in primary cells, Tie2-deficient cells are required. However, since Tie2-deficient mice die between E10.5 and E12.5 [39], we could not analyze the function of Tie2 using Tie2-deficient BM-MCps. The establishment of MCp-specific Tie2-deficient mice will foster more precise analysis of Tie2 function in MCps.

## Supporting Information

S1 Fig Tie2 and Integrin expressions on MEDMC-BRC6 transfectants. The numbers indicate ratios of mean fluorescence intensity (MFI) of anti-Tie2 mAb or anti-integrin mAb staining to that of isotype control Ab staining.
(TIF)

S2 Fig β1 integrin expressions after Ang1 treatment. MEDMC-BRC6 transfectants and mouse BM-MCps were incubated in the presence or absence of Ang1 (250 ng/mL) for 90 to 120 min. β1 integrin expressions were then analyzed by flow cytometry. Histograms of solid lines show staining of anti-β1 integrin mAbs to cells incubated with (red) and without (black) Ang1. Shaded histograms show staining of isotype control Abs to cells incubated without Ang1.
(TIF)

S3 Fig Involvement of β1 and β7 integrins in mouse BM-MCp adhesion to VCAM-1. Mouse BM-MCps were cultured with neutralizing anti-integrin Abs or control Abs (20 μg/mL each) in wells that were precoated with human IgG1 Ab or mouse VCAM-1-Fc. Adherent cells were counted by using a microscope (20 mm^2^ per well).
(TIF)

S4 Fig Ang1 treatment enhances mouse BMMC adhesion to VCAM-1 through α4β1 and α4β7 integrin. (A) β1 and β7 integrin expressions on BMMCs were analyzed by flow cytometry. (B) Mouse BMMCs were treated with or without Ang1 (250 ng/mL) and cultured in wells that were precoated with human IgG1 Ab or mouse VCAM-1-Fc. Adherent cells, as observed blue-colored, were counted by using a microscope (20 mm^2^ per well). (C) Neutralizing anti-β1 integrin Ab (20 μg/mL) or control Ab (20 μg/mL) was added under the conditions described in B. BMMCs were incubated on VCAM-1-Fc-coated wells, and adherent cells were similarly counted. (D) Neutralizing anti-β7 integrin Ab (20 μg/mL) or control Ab (20 μg/mL) was added under the conditions described in B. BMMCs were incubated on VCAM-1-Fc-coated wells, and adherent cells were similarly counted. Scale bars, 200 μm. Data show mean values ± SEM (n = 4 or 5). *p < 0.05, ***p < 0.001.
(TIF)

S1 Table Signaling motif sequences. (XLSX)

S2 Table Sample names of each cell type in GSE10246. (XLSX)

S3 Table Candidate genes in Fig 1A. The genes highlighted were expressed at more than 100 in mouse MCs (GSE10246).
(XLSX)
