# Supplementary figures and images for: Tie2 Signaling Enhances Mast Cell Progenitor Adhesion to Vascular Cell Adhesion Molecule-1 (VCAM-1) through α4β1 Integrin
Source: PLoS One. 2015 Dec 11;10(12):e0144436. doi: 10.1371/journal.pone.0144436 (PMC4687632; doi:10.1371/journal.pone.0144436)

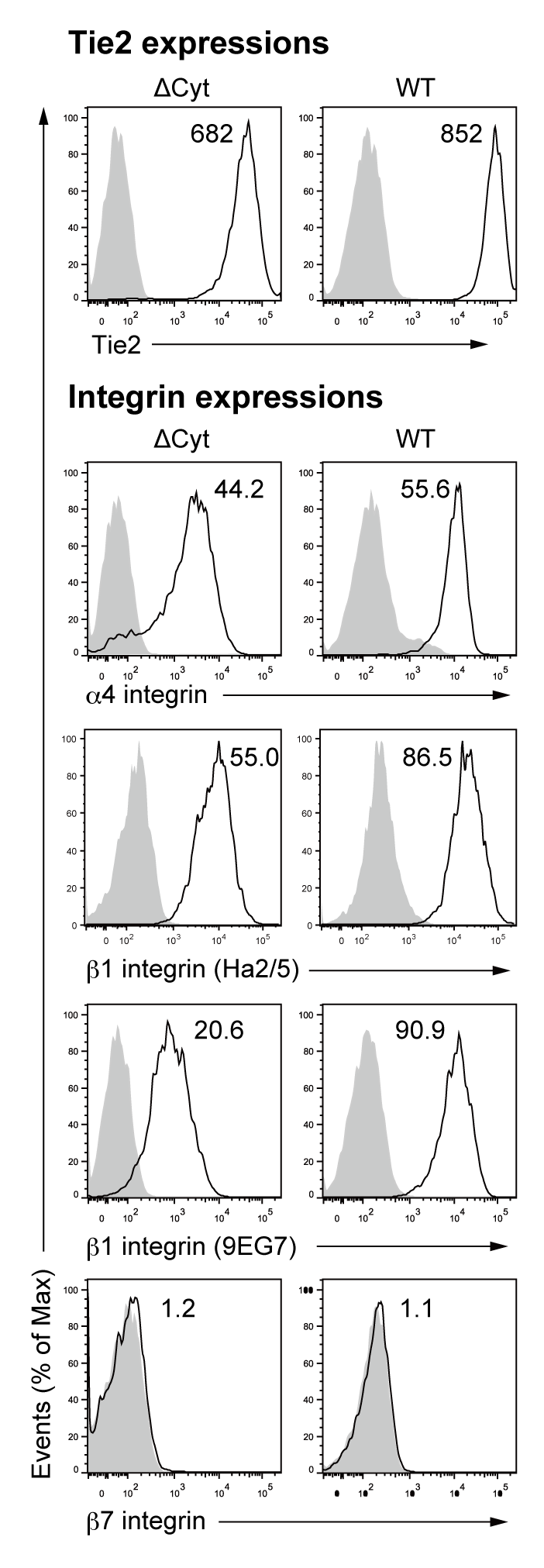

Supplement: S1 Fig — The numbers indicate ratios of mean fluorescence intensity (MFI) of anti-Tie2 mAb or anti-integrin mAb staining to that of isotype control Ab staining. (TIF) [file pone.0144436.s001.tif]

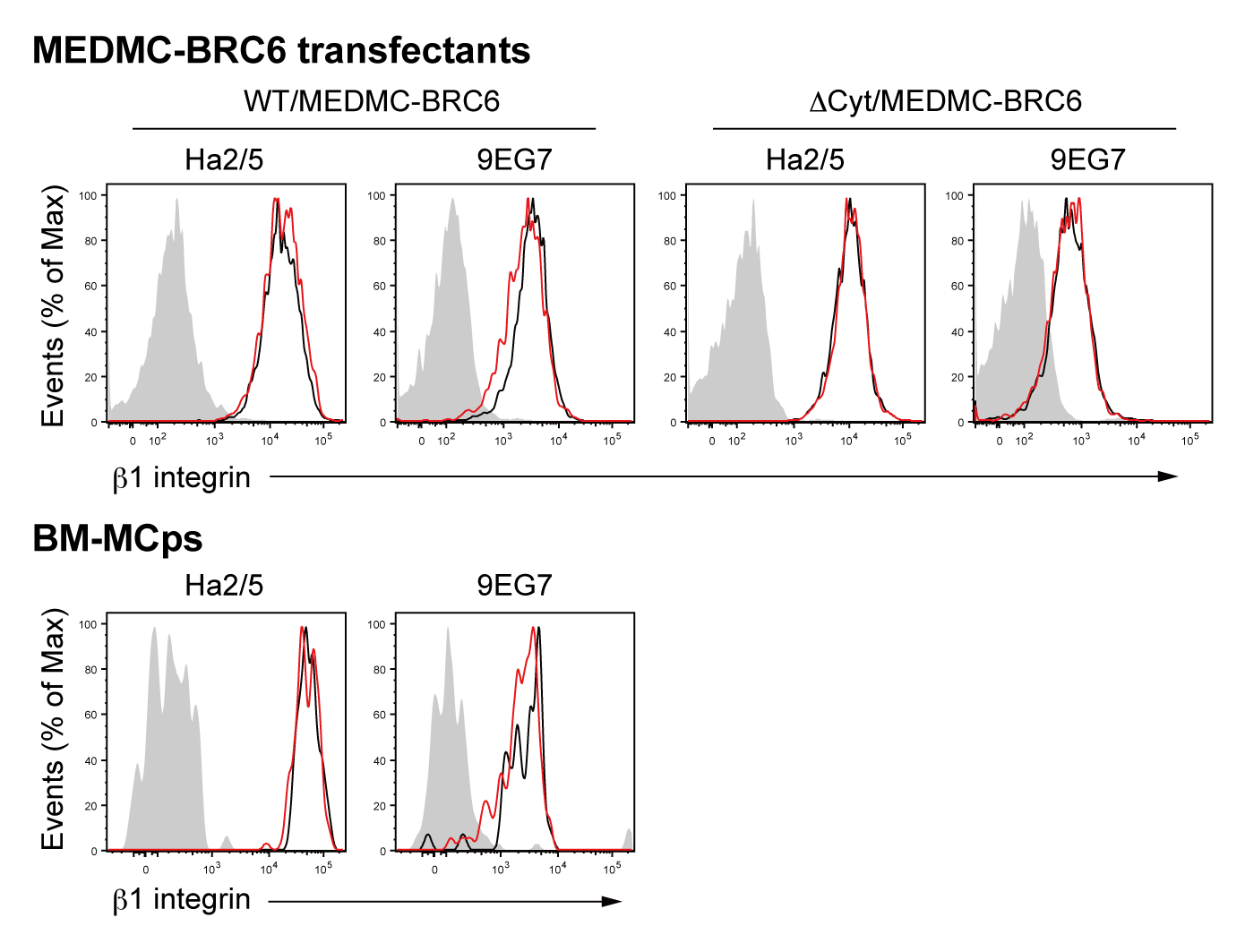

Supplement: S2 Fig — MEDMC-BRC6 transfectants and mouse BM-MCps were incubated in the presence or absence of Ang1 (250 ng/mL) for 90 to 120 min. β1 integrin expressions were then analyzed by flow cytometry. Histograms of solid lines show staining of anti-β1 integrin mAbs to cells incubated with (red) and without (black) Ang1. Shaded histograms show staining of isotype control Abs to cells incubated without Ang1. (TIF) [file pone.0144436.s002.tif]

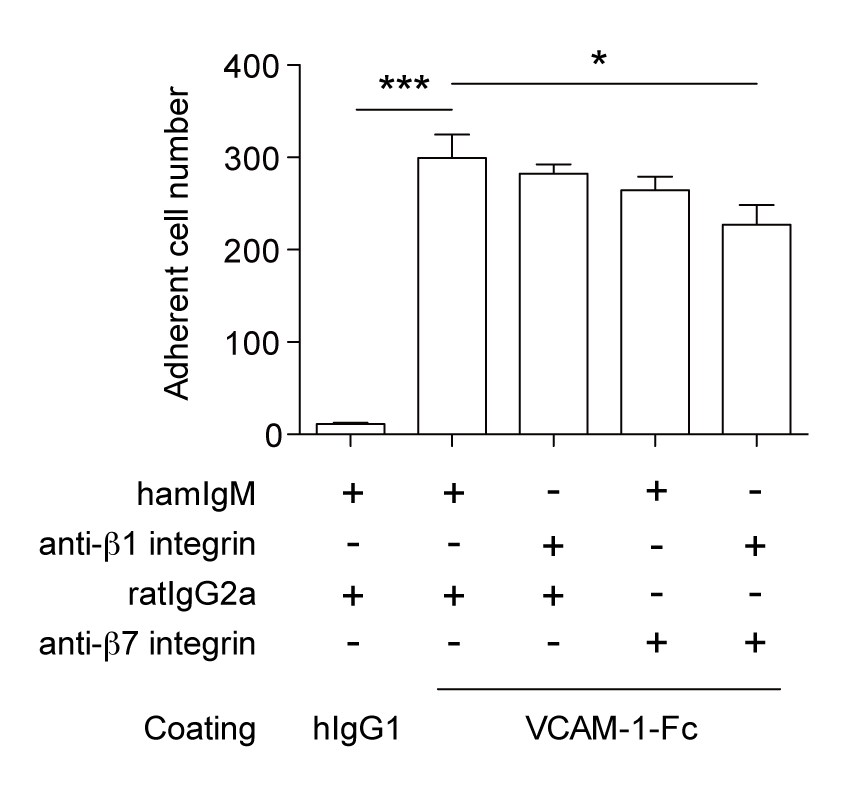

Supplement: S3 Fig — Mouse BM-MCps were cultured with neutralizing anti-integrin Abs or control Abs (20 μg/mL each) in wells that were precoated with human IgG1 Ab or mouse VCAM-1-Fc. Adherent cells were counted by using a microscope (20 mm2 per well). (TIF) [file pone.0144436.s003.tif]

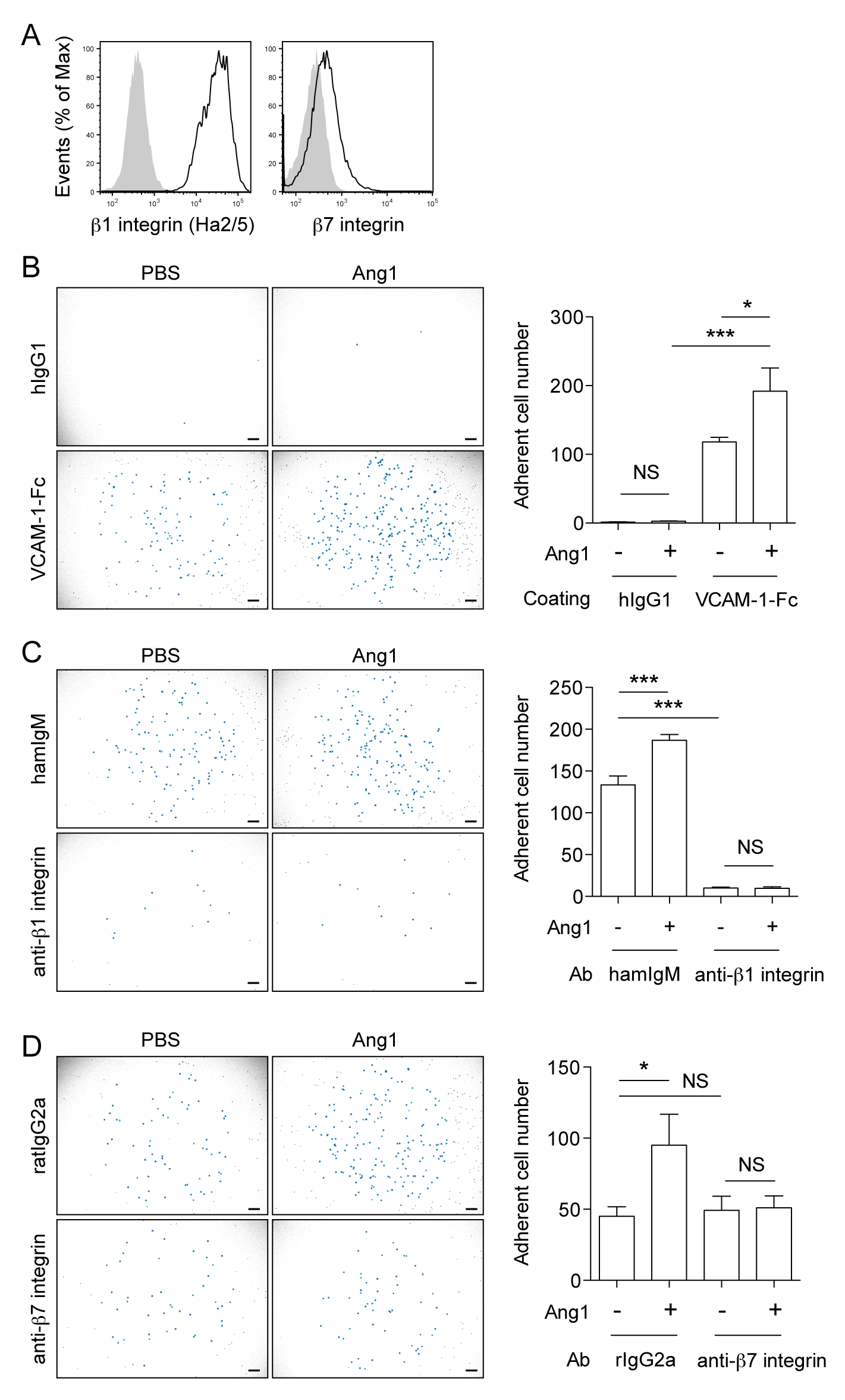

Supplement: S4 Fig — (A) β1 and β7 integrin expressions on BMMCs were analyzed by flow cytometry. (B) Mouse BMMCs were treated with or without Ang1 (250 ng/mL) and cultured in wells that were precoated with human IgG1 Ab or mouse VCAM-1-Fc. Adherent cells, as observed blue-colored, were counted by using a microscope (20 mm2 per well). (C) Neutralizing anti-β1 integrin Ab (20 μg/mL) or control Ab (20 μg/mL) was added under the conditions described in B. BMMCs were incubated on VCAM-1-Fc-coated wells, and adherent cells were similarly counted. (D) Neutralizing anti-β7 integrin Ab (20 μg/mL) or control Ab (20 μg/mL) was added under the conditions described in B. BMMCs were incubated on VCAM-1-Fc-coated wells, and adherent cells were similarly counted. Scale bars, 200 μm. Data show mean values ± SEM (n = 4 or 5). *p < 0.05, ***p < 0.001. (TIF) [file pone.0144436.s004.tif]
